# Termination layer compensated tunnelling magnetoresistance in ferrimagnetic Heusler compounds with high perpendicular magnetic anisotropy

**DOI:** 10.1038/ncomms10276

**Published:** 2016-01-18

**Authors:** Jaewoo Jeong, Yari Ferrante, Sergey V. Faleev, Mahesh G. Samant, Claudia Felser, Stuart S. P. Parkin

**Affiliations:** 1 IBM Almaden Research Center, San Jose, California 95120, USA; 2 Max-Planck Institute for Microstructure Physics, 06120 Halle (Saale), Germany; 3 Max-Planck Institute for Chemical Physics of Solids, 01187 Dresden, Germany

## Abstract

Although high-tunnelling spin polarization has been observed in soft, ferromagnetic, and predicted for hard, ferrimagnetic Heusler materials, there has been no experimental observation to date of high-tunnelling magnetoresistance in the latter. Here we report the preparation of highly textured, polycrystalline Mn_3_Ge films on amorphous substrates, with very high magnetic anisotropy fields exceeding 7 T, making them technologically relevant. However, the small and negative tunnelling magnetoresistance that we find is attributed to predominant tunnelling from the lower moment Mn–Ge termination layers that are oppositely magnetized to the higher moment Mn–Mn layers. The net spin polarization of the current reflects the different proportions of the two distinct termination layers and their associated tunnelling matrix elements that result from inevitable atomic scale roughness. We show that by engineering the spin polarization of the two termination layers to be of the same sign, even though these layers are oppositely magnetized, high-tunnelling magnetoresistance is possible.

## Abstract (web summary)

Heusler alloy thin films with a distorted tetragonal structure have potential spintronics applications given their bulk perpendicular magnetic anisotropy. Here, the authors demonstrate large perpendicular magnetic anisotropy in Mn_3_Ge thin films accompanied by negative tunnelling magnetoresistance.

## Graphical abstract

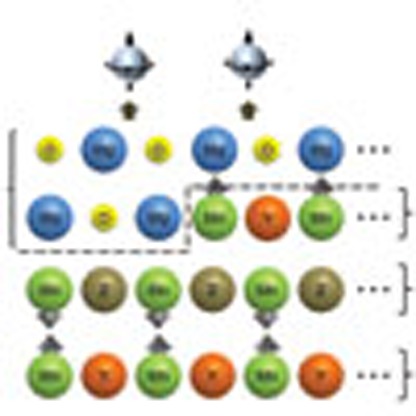

Key to the successful development of magnetic random access memory (MRAM), one of the most promising emerging non-volatile memory technologies today, are new magnetic materials for the magnetic tunnel junction (MTJ) memory elements that have sufficient stability against thermal fluctuations to sustain deeply scaled devices. The most promising magnetic materials to date are considered to be magnetic alloys formed from Co, Fe and B, in conjunction with MgO(001) tunnel barriers^1^,^2^,^3^. The magnetic electrodes must possess sufficient perpendicular magnetic anisotropy (PMA) that their magnetizations lie perpendicular to the plane of the MTJ device, since this allows for reduced currents to switch the magnetization of the electrode that forms the memory layer of the device using spin torque^3^,^4^. The PMA of Co–Fe–B layers arises from the interfaces between these layers and the tunnel barrier and/or the underlayer on which the Co–Fe–B layer is deposited. Thus, these layers must be made sufficiently thin that the interface PMA overcomes the demagnetization energy that arises from the magnetic volume and increases in proportion with the magnetic volume of the Co–Fe–B layer. In practice, this means that the PMA is too weak to overcome thermal fluctuations when the device has a critical dimension <∼20 nm in size, since the thickness of the magnetic layer has to be below that required to maintain its moment perpendicular, and, below that needed to switch the magnetic layer with reasonable current densities. Magnetic materials in which the PMA is derived from volume magnetocrystalline anisotropy are then needed. One of the most promising class of such materials are the Heusler alloys—compounds having the chemical formula X_2_YZ or X'X''YZ, wherein X, X', X” and Y are transition metals, or lanthanides (rare-earth metals), and Z is from a main group metal^5^. Some of these compounds are ferromagnetic or ferrimagnetic depending on the exchange interaction between the magnetic moments on the X and/or Y sites. Moreover, while the parent Heusler compounds are cubic and exhibit weak or no significant magnetic anisotropy, the structure of some of these compounds is found to be tetragonally distorted: due to this distortion the magnetization exhibited by these compounds may be aligned along the tetragonal axis. Thus, thin films formed from such materials may exhibit PMA due to a magnetocrystalline anisotropy associated with the tetragonally distorted structure. Some examples of such tetragonal Heusler compounds are Mn_3-*x*_Ga (ref. 6) and Mn_3_Ge (ref. 7).

Thin films of these materials have been shown to exhibit large PMA but, to date, all work on these materials has involved films that are grown epitaxially on single crystalline substrates such as SrTiO_3_(001) or MgO(001) using seed layers formed from a variety of materials but preferably Cr or Pt (refs 7, 8, 9, 10, 11). Such single crystalline substrates are not useful for MRAM applications in which the MTJs must be deposited on wires formed from polycrystalline copper, which may be covered with other layers that are also polycrystalline or amorphous.

Here we show that thin films of ferrimagnetic Mn_3_Ge with giant PMA can be grown on amorphous substrates (Si(001)/SiO_2_) using underlayers formed from TaN/IrMn_3_ (TI). The magnetic properties of these films are comparable or superior to films that we have grown under similar conditions on single crystal MgO(001) substrates using epitaxial Cr(001) underlayers. Very high magnetic anisotropy fields exceeding 7T are found. However, the tunnelling magnetoresistance (TMR) is negative and much smaller than theoretical predictions for tunnel junction devices formed with MgO tunnel barriers. We attribute this to predominant tunnelling from the lower moment Mn–Ge termination layers that are oppositely magnetized to the higher moment Mn–Mn layers. The net spin polarization of the current reflects the different proportions of the two distinct termination layers and their associated tunnelling matrix elements at the tunnel barrier interface, that result from inevitable atomic scale roughness. We have confirmed that a second perpendicularly magnetized Heusler compound, Mn_2_CuSb, which we identified by computational materials discovery methods, similarly displays small TMR, but that the sign of the spin polarization of each of the termination layers is opposite to that of their magnetization, in contrast to Mn_3_Ge. Therefore, the low TMR that we have found in Mn_3_Ge and Mn_2_CuSb, which we attribute to termination layer compensation, is not an inherent property of ferrimagnetic Heuslers. The spin polarization of the two termination layers can be engineered to be of the same sign even though these layers are oppositely magnetized, therefore providing a path to low magnetization electrodes with high TMR for high density spin transfer torque MRAM applications.

## Results

### Growth of highly textured Mn_3_Ge on amorphous substrates

Highly textured, polycrystalline and tetragonal Mn_3_Ge films were grown by either ion-beam deposition or d.c.-magnetron sputtering in an ultra-high vacuum chamber with a base pressure of ∼4 × 10^−10^ torr on Si(001) substrates covered with 250 Å of amorphous SiO_2_. We find that seed layers formed from bilayers of TaN/IrMn_3_ that are first deposited on the SiO_2_ induce (001) textured Mn_3_Ge films that are tetragonally distorted. IrMn_3_ is known to have a L1_2_ structure that is cubic, and which matches one of the sublattices of the structure that Mn_3_Ge is known to form. Moreover, the lattice mismatch between IrMn_3_ and Mn_3_Ge is <1% (ref. 12). The TaN layer that is grown by reactive sputtering, promotes the growth of (001) textured IrMn_3_, since fcc-IrMn_3_ favours the (111) out-of-plane orientation when grown directly on amorphous SiO_2_ surface (Supplementary Fig. 1 and Supplementary Note 1). However, in contrast to theoretical predictions of giant values of TMR^6^,^13^,^14^ for MTJs using Mn_3_Ge electrodes we find much smaller values experimentally, which we attribute to compensation in the tunnelling spin current polarization from atomic layer variations of the electrode surface termination at the tunnel barrier interface. We propose that this is an inevitable consequence of ferrimagnets with layer-by-layer alternation of magnetization, when the spin polarization of these layers compensates each other. We confirm this conjecture by preparing MTJs using another ferrimagnetic Heusler compound, Mn_2_CuSb, which also shows high PMA but yet very small TMR.

The structural and magnetic properties of the Mn_3_Ge films depend sensitively on its composition and atomic order. The latter is strongly influenced by the deposition temperature and subsequent anneal conditions, which also affect the smoothness of the Mn_3_Ge film. To achieve optimal MTJ performance the electrode must be atomically smooth. We find that the root mean square (r.m.s.) roughness of the Mn_3_Ge film, *r*_r.m.s._, measured using atomic force microscopy, increases significantly when the growth temperature (*T*_G_) exceeds modest temperatures of just ∼200 °C, but higher growth temperatures are needed to sustain the Heusler structure, as measured from X-ray diffraction (Supplementary Fig. 2 and Supplementary Note 2). Thus, we find that an optimal growth method, which includes a three-step process for the Mn_3_Ge electrode, in which an initial 20 Å Mn_3_Ge layer is grown at 450 °C, followed by a thicker Mn_3_Ge layer deposited at *T*_G_=150 °C, with a final *in situ* anneal at 450 °C for 1–2 h in vacuum, gives smooth films (*r*_r.m.s._∼3 Å) with high PMA. During the annealing step of Mn_3_Ge films, there is a substantial inter-diffusion between IrMn_3_ and Mn_3_Ge layers (Supplementary Fig. 3), which causes deterioration of the magnetic properties of Mn_3_Ge. We discovered that this interdiffusion can be prevented by using a thin 10–20 Å TaN barrier between IrMn_3_ and Mn_3_Ge layers, as revealed by electron energy loss spectroscopy measurements (Supplementary Note 3). Thus, the preferred underlayer is formed from TaN/IrMn_3_/TaN (TIT). We note that a single TaN underlayer gives much poorer quality Mn_3_Ge layers.

**Figure 1:**
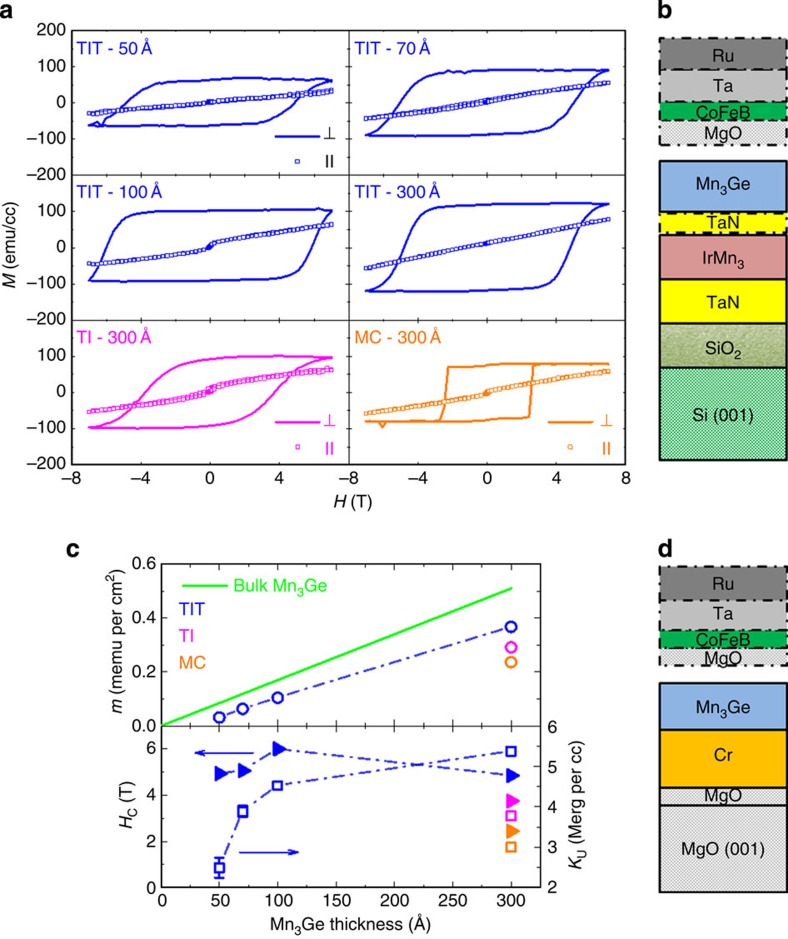
Mn_3_Ge Heusler films with giant perpendicular magnetic anisotropy. (**a**) Magnetization versus field hysteresis loops of Mn_3_Ge films grown on Si(001)/SiO_2_ substrate with TI (magenta) and TIT (blue) underlayers and MgO(001) substrate with MC (orange) underlayers. TI (TIT) have the following structure: Si(001)/SiO_2_/200 Å TaN/200 Å IrMn_3_ (/10 Å TaN) while MC has the following structure: MgO(001)/20 Å MgO/400 Å Cr. For the TIT films, the thickness of the Mn_3_Ge layer, deposited using the three-step process, was varied. Out of plane (in-plane) *M* vs. *H* loops are shown as solid lines (open squares). **b** and **d** are schematics of the MTJ structures grown on Si(001)/SiO_2_ and MgO(001) substrates, respectively. In some cases a TaN diffusion barrier layer was used as indicated by a dashed line. For characterization of structural, topographical and magnetic properties of the Mn_3_Ge films, a 30 Å Ta film was used as a capping layer instead of the upper layers shown within the dotted lines. (**c**) Magnetic moment *m*, coercive field *H*_C_ (solid triangles) and uniaxial magnetic anisotropy constant *K*_U_ (empty squares), extrapolated from Fig. 1a, versus Mn_3_Ge thickness. The green straight line shows the calculated moment of bulk D0_22_-Mn_3_Ge (ref. 15). As shown in Fig. 1a, the Mn_3_Ge magnetization cannot be saturated in-plane using the available magnetic field (7 T); thus, *H*_eff_ is a lower bound.

Figure 1 compares the magnetic properties of Mn_3_Ge films with varying thickness grown on amorphous substrates using the three-step process and on a crystalline MgO(001) substrate with a Cr seed layer (MC). Excellent PMA is observed in all cases but the highest coercive and anisotropy fields are found for structures grown on the TIT underlayer. Coercive fields of 6T and anisotropy fields exceeding 7T are found. Figure 1c summarizes the magnetic moment *m*, coercivity *H*_C_ and uniaxial magnetic anisotropy *K*_U_ for these films. Values of *m* for Mn_3_Ge films grown on TIT underlayers are close to those theoretically predicted for bulk Mn_3_Ge (ref. 15), but *m* is significantly lowered by ∼15–35% for Mn_3_Ge films grown using TI underlayers or a MC single crystal substrate. For the latter film, we also find that the magnetic anisotropy *K*_U_ is substantially lower: we attribute this to the large lattice mismatch (∼7%) between Cr and Mn_3_Ge.

### Tunnelling magnetoresistance of Mn_3_Ge-based MTJ devices

MTJ devices were fabricated using standard lithographic techniques from film stacks whose structures are illustrated in Fig. 1b. The reference electrode was formed from the Mn_3_Ge Heusler compound, and the free electrode from an ultrathin layer of CoFeB with a composition of 20:60:20. Before patterning, these films were post-annealed at 350 °C for 60 min in a high-vacuum chamber using an applied magnetic field of 1T directed out of the plane of the sample. Devices with sizes of 1 × 2 μm^2^ and ∼30 nm in diameter were fabricated by optical lithography and e-beam lithography, respectively. Only the free layer was patterned to define the junction size while the reference layer was not patterned.

**Figure 2:**
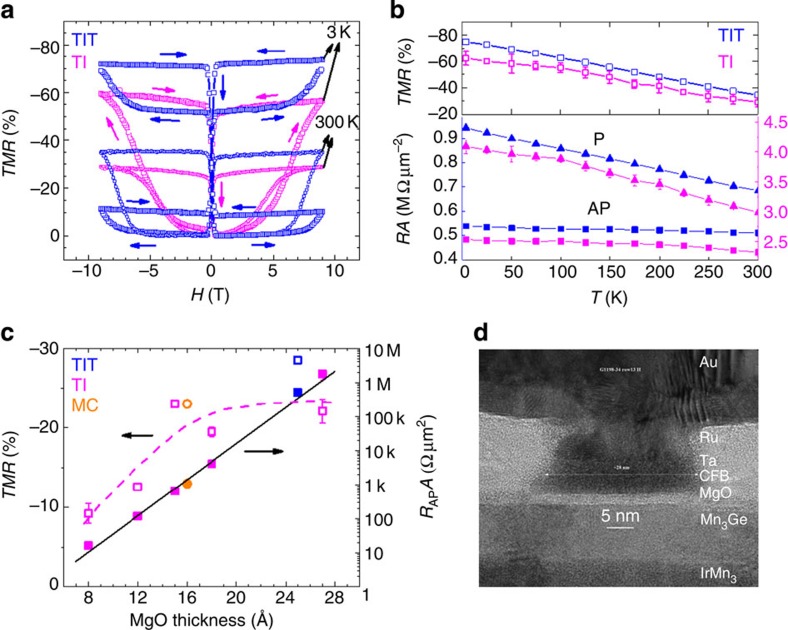
Characteristics of Mn_3_Ge-based magnetic tunnel junctions. (**a**) TMR versus *H* (perpendicular to the device) measured at 300 K (smaller squares) and 3 K (bigger squares) for MTJ devices grown using TI (magenta) and TIT (blue) underlayers. For the TIT junction, two sets of data were measured at 3 K (blue bigger squares) after cooling down the device from 300 K in a magnetic field of +9 and −9 T, respectively. These data are mirror images of each other, as can be seen in the figure. All the other measurements were performed without field cooling. (**b**) Temperature dependence of TMR, and *R*_P_*A* and *R*_AP_*A*. (**c**) MgO thickness dependence of TMR (open symbols) and *R*_AP_*A* product (solid symbols), averaged over >20 devices. Solid and dashed lines are guides to the eye for *R*_AP_*A* and TMR, respectively. *R*_AP_*A* scales exponentially with barrier thickness. (**d**) HRTEM image of an MTJ device ∼27 nm in size, with the structure: Si/250 Å SiO_2_/200 Å TaN/200 Å IrMn_3_/300 Å Mn_3_Ge/15 Å MgO/15 Å CoFeB/50 Å Ta/50 Å Ru. HRTEM, high-resolution transmission electron microscopy.

Figure 2a compares TMR versus perpendicular magnetic field *H* measured at 300 K (smaller squares) and 3 K (larger squares) for patterned MTJ devices (1 × 2 μm^2^) using TI and TIT underlayers. In each case high applied magnetic fields (±9 T) are needed to align the magnetic moments of the Mn_3_Ge and CoFeB layers parallel to each other (P state) because of the giant uniaxial anisotropy of Mn_3_Ge. The junction resistance is higher in the P state compared with the antiparallel (AP) state, obtained when the CoFeB moment switches close to zero field. Thus, the TMR ([(*R*_AP_-*R*_P_)/*R*_AP_] × 100) is negative with values of∼−35% at 300 K and∼−74% at 3 K, where *R*_P_ and *R*_AP_ are the junction resistances in the P and AP states, respectively. These are the highest values of TMR reported to date in perpendicularly magnetized MTJ devices using a tetragonally distorted Heusler compound as a magnetic electrode. Nonetheless, these values are much smaller than those predicted by density functional theory (DFT) calculations^14^, as discussed below.

For a given MTJ device *R*_AP_ barely changes, while *R*_P_ increases monotonically as *T* decreases, resulting in higher TMR at low temperatures (Fig. 2b). These properties, as well as the dependence of the resistance-area product *R*_AP_*A* and TMR on the barrier thickness (Fig. 2c) are characteristic of a high quality tunnel barrier. A cross-sectional high-resolution transmission electron microscopy image of a typical MTJ device with a width of 27 nm, shown in Fig. 2d, illustrates the high quality of the structure and the device patterning.

Notwithstanding the exceptionally high PMA values exhibited by polycrystalline Mn_3_Ge films the surprisingly low TMR values lessens their potential importance for MTJ devices. We attribute the low TMR, as discussed below, to their ferrimagnetic structure. This limitation could be overcome by identifying Heusler compounds that display high PMA, and which are ferromagnetic.

### Discovery of tetragonal Heuslers by computational methods

To identify potential candidate tetragonal materials we used computational materials discovery methods to calculate the structure and electronic properties of several hundred Heusler compounds (with X=Mn, Fe, Co, Ni, Cu, Ru, Rh, Pd; Y=Sc, Mn, Fe, Co, Ni, Cu, Ru, Rh, Pd, Os, Ir, Pt; and Z=Al, Si, Ga, Ge, In, Sn, Sb) for both the regular and inverse structures and for ferri- and ferromagnetic configurations. Surprisingly, ∼40% of these compounds are calculated to be tetragonal in their ground state. Rank ordering these tetragonal compounds according to the combination of the energy difference between the tetragonal and cubic structures and that between the inverse and regular structures, we identified ∼30 possible candidate tetragonal materials, where the average energy difference exceeded ∼0.3 eV per formula unit. Of these, we prepared eight in thin film form using the same buffer layers and deposition conditions as discussed above. Half of these compounds showed a tetragonal structure and two showed excellent PMA properties, namely, Mn_2_CuSb and Rh_2_CoSb. In the other cases, we rationalize that the theoretical predictions were not fulfilled due to atomic disorder on the X and Y sites. This is particularly the case where X and Y have similar chemical properties or atomic size.

**Figure 3:**
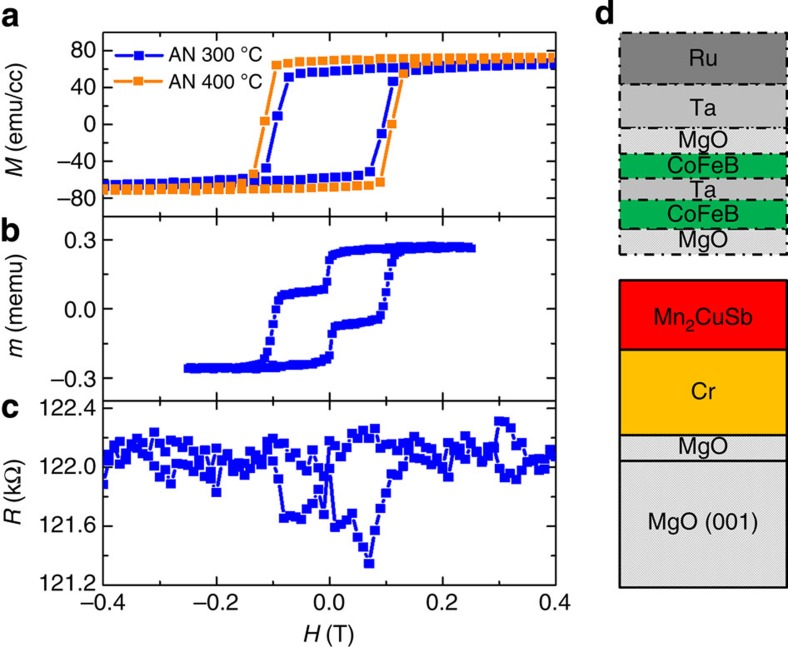
Magnetics properties and TMR of Mn_2_CuSb Heusler alloy. (**a**) Out-of-plane *M* versus *H* hysteresis loops, at 300 K, of 300 Å thick Mn_2_CuSb films grown on MC MgO(001) substrate at room temperature and post annealed at 300 °C (blue) and 400 °C (orange) for 30 min. The anneal treatments were carried out in a high-vacuum anneal furnace, in an applied magnetic field of 1 T (out-of-plane direction) for 30 min. (**b**) Out-of-plane *M* vs. *H* hysteresis loop of an MTJ film measured before lithography patterning. (**c**) Two-terminal junction *R* vs. *H* loop of a patterned MTJ device (1 × 2 μm^2^ size). (**d**) Schematic of a Mn_2_CuSb-based MTJ, with the structure: MgO(001)/20 Å MgO/400 Å Cr/300 Å Mn_2_CuSb/25 Å MgO/14 Å CoFeB/4 Å Ta/7 Å CoFeB/7 Å MgO/50 Å Ta/100 Å Ru. The Mn_2_CuSb electrode was deposited at ambient temperature and *in situ* annealed at 300 °C before the MgO barrier deposition. The entire stack was then post annealed at 325 °C for 30 min.

Similar magnetic properties were found for Mn_2_CuSb and Rh_2_CoSb films grown using either TI underlayers (Supplementary Fig. 4 and Supplementary Note 4) or an MC crystalline substrate but the latter films were typically smoother and better suited for MTJ studies. Results are shown in Fig. 3 for Mn_2_CuSb. Square magnetic hysteresis loops consistent with PMA are found. The calculated lowest energy configuration of Mn_2_CuSb is a tetragonal ferromagnetic regular Heusler with a moment of 5.4 *μ*_B_ per formula unit (Table 1). Although we find that the deposited films are tetragonal (Supplementary Fig. 5 and Supplementary Note 5), in agreement with the calculations, the measured *c*/*a* ratio is much smaller in the films (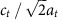 ∼1.1) than that predicted for a fully ordered Mn_2_CuSb bulk compound (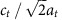
∼1.4), and thus only slightly distorted from the cubic phase. Moreover, the magnetization of the films is much too low—∼0.4 *μ*_B_ per formula unit—to be consistent with the predicted ferromagnetic state, as is the TMR, as we discuss below. We rationalize these findings by the likelihood of chemical disorder within the deposited films. We note that the calculations predict that for the cubic phase the structure will be the inverse structure with a ferrimagnetic ordering, with a low moment (Table 1).

MTJ devices were prepared from Mn_2_CuSb films with the layer stack shown in Fig. 3d and the magnetic hysteresis loop shown in Fig. 3b. The latter shows clear independent switching of the free layer (CoFeB) and the reference layer (Mn_2_CuSb) but TMR curves measured on 1 × 2 μm^2^ size devices show only very small TMR values of∼−1% (Fig. 3c).

### Termination-layer dependent tunnelling spin polarization

**Figure 4:**
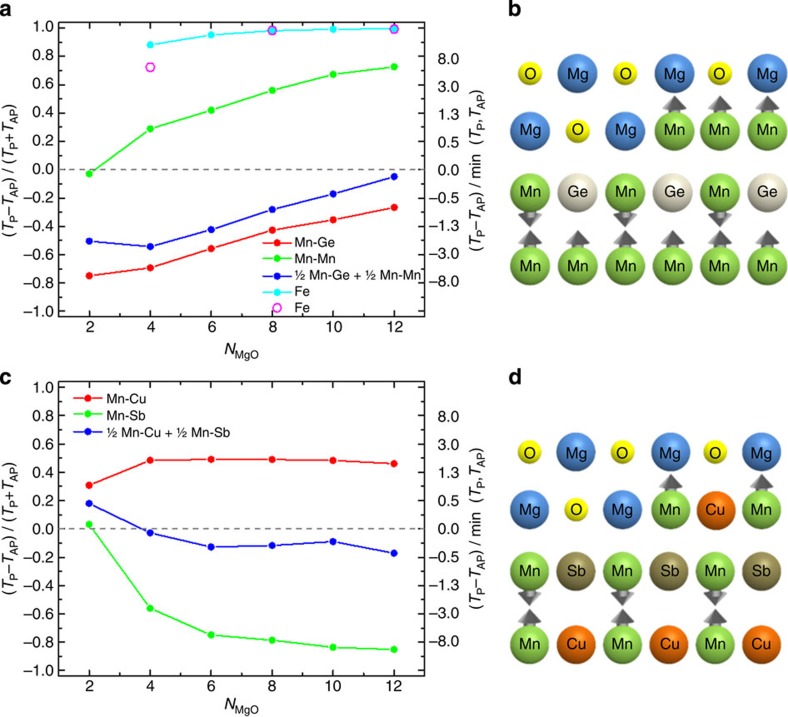
Theoretical calculations of tunneling magnetoresistance. (**a**) TMR for Mn_3_Ge/MgO/Fe and Fe/MgO/Fe MTJs versus *N*_MgO_. TMR for Mn_3_Ge/MgO/Fe MTJ with Mn–Mn termination is shown by green line, with Mn–Ge termination by red line and for device with steps (half of the device area has Mn–Mn termination and half of the area has Mn–Ge termination) is shown by blue line. The TMR for Fe/MgO/Fe MTJ calculated in this work by tight-binding linear muffin-tin orbital (TB–LMTO) method is shown by cyan line and calculated by layer Korringa-Kohn-Rostoker (KKR) method (ref. 22) is shown by pink circles. (**b**) Schematic of atomic step between two distinct terminations with opposite magnetic moments for Mn_3_Ge. (**c**) The TMR for Mn_2_CuSb/MgO/Fe MTJ with Mn–Sb termination is shown by green line, with Mn–Cu termination by red line and for device with steps (half of the device area has Mn–Cu termination and half of the area has Mn–Sb termination) is shown by blue line. (**d**) Schematic of atomic step between two distinct terminations with opposite magnetic moments for Mn_2_CuSb. Note that in **a** and **c** the TMR for the termination with the larger magnetic moment is shown in green for both Mn_3_Ge and Mn_2_CuSb. In **b** and **d** the direction of the magnetic moments shown for the different termination layers for the different steps corresponds to the sign of the TMR for that termination layer (that is, arrow pointing up (down) corresponds to positive (negative) TMR).

We performed *ab initio* calculations of the electronic structure and transport properties of Mn_3_Ge/MgO/Fe and Mn_2_CuSb/MgO/Fe MTJs (see Methods for details of the calculations). Note that we use bcc Fe rather than CoFeB that is used in the experiments to simplify the calculations. The TMR for Mn_3_Ge/MgO/Fe MTJs is shown in Fig. 4a as function of the number of MgO layers, *N*_MgO_, for *N*_MgO_≥2. Two definitions of TMR are shown: (*T*_P_−*T*_AP_)/min(*T*_P_,*T*_AP_), that can vary from −∞ to ∞, (that we use for the experimental data) and (*T*_P_−*T*_AP_)/(*T*_P_+*T*_AP_), that can vary from −1 to 1. Here *T*_P_ and *T*_AP_ are the transmission functions (calculated at zero bias voltage) corresponding to the P and AP states, respectively. The most important result is that the TMR depends sensitively on the atomic configuration of the termination layer in the Mn_3_Ge adjacent to the MgO tunnel barrier. The calculation gives magnetic moments of +4.08 and −3.12 *μ*_B_ for the Mn–Mn and Mn–Ge termination layers, respectively. The *TMR* has opposite signs for the two termination layers, Mn–Mn and Mn–Ge, increasing in magnitude with *N*_MgO_ for Mn–Mn but decreasing in magnitude for Mn–Ge. These results can be understood from the layer dependent, spin-dependent density of states calculated for the bulk electronic structure of Mn_3_Ge, and the well understood symmetry spin-filtering properties of the MgO/Fe interface. The spin polarization of the native termination layers is negative in both cases but much bigger for Mn–Ge (see Supplementary Fig. 6 and Supplementary Note 6 for more details). A Brillouin zone filtering effect^16^ arising from the Mn_3_Ge/MgO interface tends to make the TMR positive for both terminations as the MgO layer thickness is increased. Thus, the TMR is negative for Mn–Ge but positive for Mn–Mn for intermediate MgO thicknesses that are of interest experimentally. The balance between the native spin polarization and the Brillouin zone filtering not only accounts for the dependence of the TMR on *N*_MgO_ and the termination layer but also the bias voltage (Supplementary Fig. 7 and Supplementary Note 7).

These calculations give a natural explanation for the low TMR values found experimentally using Mn_3_Ge electrodes. Even though the Mn_3_Ge/MgO interface is very smooth (Fig. 2d) inevitably there will be atomic scale fluctuations in the morphology of the Mn_3_Ge layer that gives rise to regions with Mn–Mn and Mn–Ge terminations, due to the fundamental underlying structure of the Heusler compound (see illustration in Fig. 4b). The simplest way to model such fluctuations is to average the transmission functions over the different terminations (separately for P and AP states), assuming that the MgO thickness is the same across the device. The TMR calculated from this simple model with an assumption of equal areas occupied by Mn–Ge and Mn–Mn terminations is shown in Fig. 4a. The calculations give a negative TMR since both *T*_P_ and *T*_AP_ for the Mn–Ge termination are larger than those for the Mn–Mn termination (for all *N*_MgO_ considered–ranging from 2 to 12). The negative TMR is consistent with our experimental measurements. Note that due to the large 10.5% lattice mismatch between MgO and Mn_3_Ge the Brillouin zone filtering effect, which critically depends on the existence of the well-defined 2D Brillouin zone of the Mn_3_Ge/MgO interface, is likely more suppressed in actual devices as compared with the large negative spin polarization effect that is less sensitive to the existence of the 2D Brillouin zone. Thus, both ideal crystal theoretical simulations (Fig. 4a) and non-ideal crystal arguments predict negative TMR for the Mn_3_Ge/MgO/Fe system, in agreement with experimental results (at low temperatures the TMR=−75% for ∼27 Å thick MgO: Fig. 2b). We note that the surface energy of the different terminations will very likely not be identical. This could lead to the formation of a more complex surface structure but kinetic considerations will constrain the surface beyond that of the equilibrium structure.

The calculated TMR versus *N*_MgO_ for Mn_2_CuSb/MgO/Fe MTJs, where the Mn_2_CuSb has a tetragonal ferrimagnetic inverse Heusler structure, with Mn–Sb and Mn–Cu terminations at the MgO interface are compared in Fig. 4c. Note that the calculated magnetic moment of the Mn–Sb layer (3.2 *μ*_B_) is larger than that for the Mn–Cu layer (−2.8 *μ*_B_). Interestingly, the tunnelling spin polarization from the native termination layers, that is, in the limit of *N*_MgO_→0, is of the opposite sign to the magnetic moment direction of these layers, whereas for Mn_3_Ge the spin polarization is negative for both terminations (Supplementary Fig. 8 and Supplementary Note 8). But for the range of MgO thicknesses of interest for technologically relevant MTJs (2<*N*_MgO_≤12), tunnelling spin polarization is parallel and antiparallel, to the magnetic moment of the respective termination layer for Mn_3_Ge and Mn_2_CuSb, respectively. Moreover, there is no significant Brillouin zone filtering effect for Mn_2_CuSb/MgO. In this case the dependence of the TMR on *N*_MgO_ is due to the symmetry spin filtering effect from MgO/Fe, as can be seen from Fig. 4c by comparing the calculated TMR for Mn_2_CuSb/MgO/Fe, for both terminations, with that for Fe/MgO/Fe. For the Mn_2_CuSb/MgO/Fe MTJ a negative TMR is predicted for the termination layer with the largest magnetic moment (Mn–Sb) whereas for Mn_3_Ge/MgO/Fe the opposite is the case. The TMR calculated for an MTJ with equal areas of Mn–Cu and Mn–Sb terminations is shown in Fig. 4c. The resulting TMR is significantly reduced due to the cancellation of contributions from the different terminations and has an overall negative sign (for *N*_MgO_>2). The negative sign of TMR and its small value agrees with our experimental findings.

## Discussion

In summary, we have shown a method to grow highly textured ferrimagnetic Heusler films with large perpendicular anisotropy on amorphous substrates, thereby opening a path to their potential use for many applications such as magnetic recording media and rare-earth-free hard magnets. However, we find that the TMR is strongly influenced by unavoidable atomic steps at the tunnel barrier interface. When the two termination layers have opposite tunnelling spin polarizations they compensate one another leading to low TMR, as we have shown is the case for both Mn_3_Ge and Mn_2_CuSb. However, the tunnelling spin polarization can be aligned either parallel, as in the case of Mn_3_Ge, or antiparallel, as in the case of Mn_2_CuSb, to the magnetization of the termination layers. We conclude that the most interesting and technologically useful ferrimagnetic Heusler materials will have termination layers with the same tunnelling spin polarization, that is, for one termination layer the tunnelling spin polarization is parallel to the magnetization, and for the other it is the opposite. Such materials can be identified by the use of computational techniques.

## Methods

### Mn_3_Ge and Mn_2_CuSb film deposition and characterization

Mn_3_Ge and Mn_2_CuSb films were deposited by d.c.-magnetron sputtering or ion-beam deposition at temperatures that were varied from ambient to 550 °C. Ta_*x*_N films were deposited with different Ar/N_2_ ratios by reactive magnetron sputtering from a Ta target, and 200 Å IrMn_3_ films were deposited using ion beam sputtering from a IrMn_3_ target. Ta_*x*_N and IrMn_3_ layers were grown at room temperature. Film compositions were measured by Rutherford back scattering measurements. X-ray diffraction measurements were carried out using a Bruker GADDS or a Bruker D8 Discover system. For the Ar/N_2_ ratios within the range 95/5–75/25, all IrMn_3_ films on Ta_*x*_N had a preferential orientation of (001). IrMn_3_ film grown on pure Ta underlayer showed a (111) orientation. Atomic force microscopy film characterization was made with a Bruker Icon Dimension with ScanAsyst system. High-resolution transmission electron microscopy and electron energy loss spectroscopy studies were made using a JEOL ARM 200F with a Cold-FEG source operated at 200 keV. Magnetic properties were measured at 300 K using a quantum design superconducting quantum interference device vibrating sample magnetometer (SQUID-VSM) in magnetic fields of up to ±7 T in both in-plane and out-of-plane directions. Uniaxial magnetic anisotropy *K*_U_ values were calculated from *K*_U_=*H*_eff_ × *M*_S_/2+2*πM*_S_^2^ (*H*_eff_ being the effective magnetic field and *M*_S_ the saturation magnetization).

### Magnetic tunnel junction fabrication and characterization

MTJ devices were encapsulated in Al_2_O_3_. Electrical contacts were formed from 50 Å Ru/650 Å Au. TMR of the patterned devices was measured using a Quantum Design DynaCool physical property measurement system and a custom-built probe station with Keithley source metres 2,602 and 2,400. For fast evaluation of TMR, *R*_AP_ and *R*_P_ values in Fig. 2c were measured at +0.3 T and −0.3 T, respectively, instead of sweeping the magnetic field from ±9  T.

### Calculation details

The electronic structure and transmission functions of Mn_3_Ge/MgO/Fe, Mn_2_CuSb/MgO/Fe and Fe/MgO/Fe MTJs were calculated using a tight-binding linear muffin-tin orbital method in the atomic sphere approximation with the local density approximation of DFT for the exchange-correlation energy^17^,^18^. For the Mn_3_Ge/MgO/Fe MTJ, the in-plane lattice constant was fixed to the experimental lattice constant *a* of bulk tetragonal Mn_3_Ge (*a*=3.816 Å and *c*=7.261 Å (ref. 19)). For Mn_2_CuSb/MgO/Fe the in-plane lattice constant was fixed to the calculated lattice constant *a* of the Mn_2_CuSb tetragonal inverse ferrimagnetic phase *a*=3.95 Å (Table 1).

Relaxed positions of atoms at the Mn_3_Ge/MgO and Mn_2_CuSb/MgO interfaces (for all possible terminations) were determined using the VASP molecular dynamic program^20^. The O-top configuration was found to be the most stable configuration (as compared with Mg-top and hollow) for both terminations at the Mn_3_Ge/MgO interface (in agreement with ref. 14), and for both terminations at the Mn_2_CuSb/MgO interface. For Fe/MgO interface the atomic positions from ref. 21 were used.

## Additional information

**How to cite this article:** Jeong, J. *et al*. Termination layer compensated tunnelling magnetoresistance in ferrimagnetic Heusler compounds with high perpendicular magnetic anisotropy. *Nat. Commun.* 7:10276 doi: 10.1038/ncomms10276 (2016).

## Supplementary Material

Supplementary Information Supplementary Figures 1-8, Supplementary Notes 1-8 and Supplementary References.

## Figures and Tables

**Table 1:** Calculated magnetic and structural properties of Mn_2_CuSb.

Heusler structure	Coupling of the magnetic moments of two Mn atoms	*E*_tot_ in cubic phase (eV)	Lattice constant *a*_*c*_ in cubic phase (Å)	Magnetic moment in cubic phase (*μ*_B_)	*E*_tot_ in tetragonal phase (eV)	Lattice constant *a*_*t*_ in tetragonal phase (Å)	Lattice constant *c*_*t*_ in tetragonal phase (Å)	Magnetic moment in tetragonal phase (*μ*_B_)
Regular	ferro	−25.111	6.23	6.4	−25.569	3.86	7.80	5.4
Regular	ferri	−25.107	6.28	0	No stable solution found.			
Inverse	ferro	−24.840	6.12	4.3	−25.292	3.83	8.19	6.1
Inverse	ferri	−25.176	6.22	1.0	−25.272	3.95	7.82	0.4

DFT, density functional theory; *E*_tot_, total energy.

Calculated by the DFT-based VASP^20^ program: lattice parameters and magnetic moments for different configurations of Mn_2_CuSb Heusler alloy (*T*=0 K).
