# Supplementary material for: Termination layer compensated tunnelling magnetoresistance in ferrimagnetic Heusler compounds with high perpendicular magnetic anisotropy
Source: Nat Commun. 2016 Jan 18;7:10276. doi: 10.1038/ncomms10276 (PMC4735609; doi:10.1038/ncomms10276)
Supplement: Supplementary Information — Supplementary Figures 1-8, Supplementary Notes 1-8 and Supplementary References. [file ncomms10276-s1.pdf]

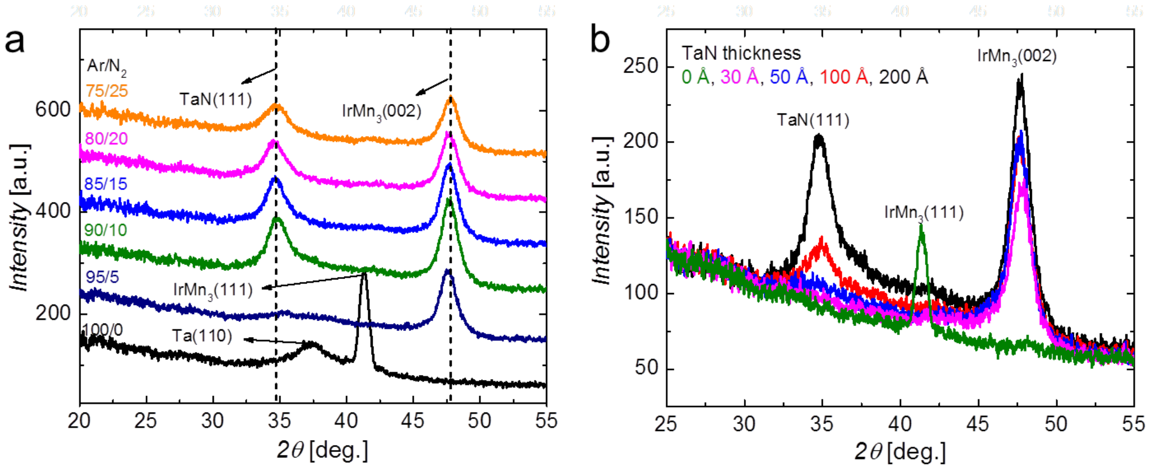

Supplementary Figure 1. XRD out-of-plane *θ-2θ* scans for TaN/ $IrMn_3$ layers grown on Si/ SiO_2 substrates. **a** XRD scans for 200 Å Ta_xN grown at various Ar/N_2 ratios. **b** *θ-2θ* scans for different TaN thicknesses.

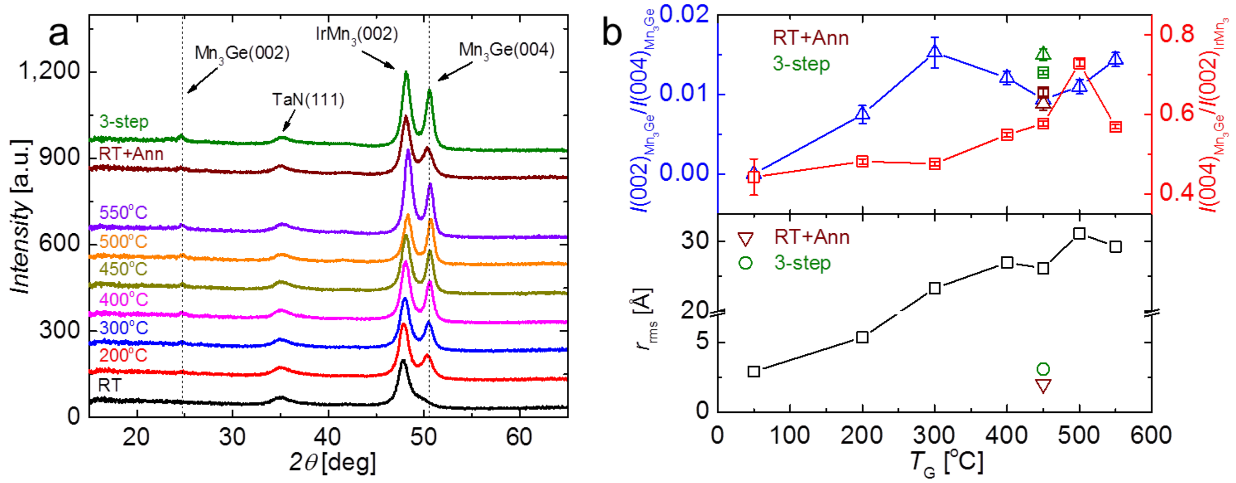

Supplementary Figure 2. Structural and topographical properties of Mn_3Ge films on TI.

a XRD measurements of 300 Å Mn_3Ge films grown at different growth temperatures on Si(001)/ SiO_2 substrates using TI underlayers and capped with 30 Å Ta. Top two curves, shown for comparison, represent RT deposition with in-situ anneal (brown) and 3-step process deposition (olive). **b** Dependence of chemical ordering and r_{rms} of Mn_3Ge films on T_G.

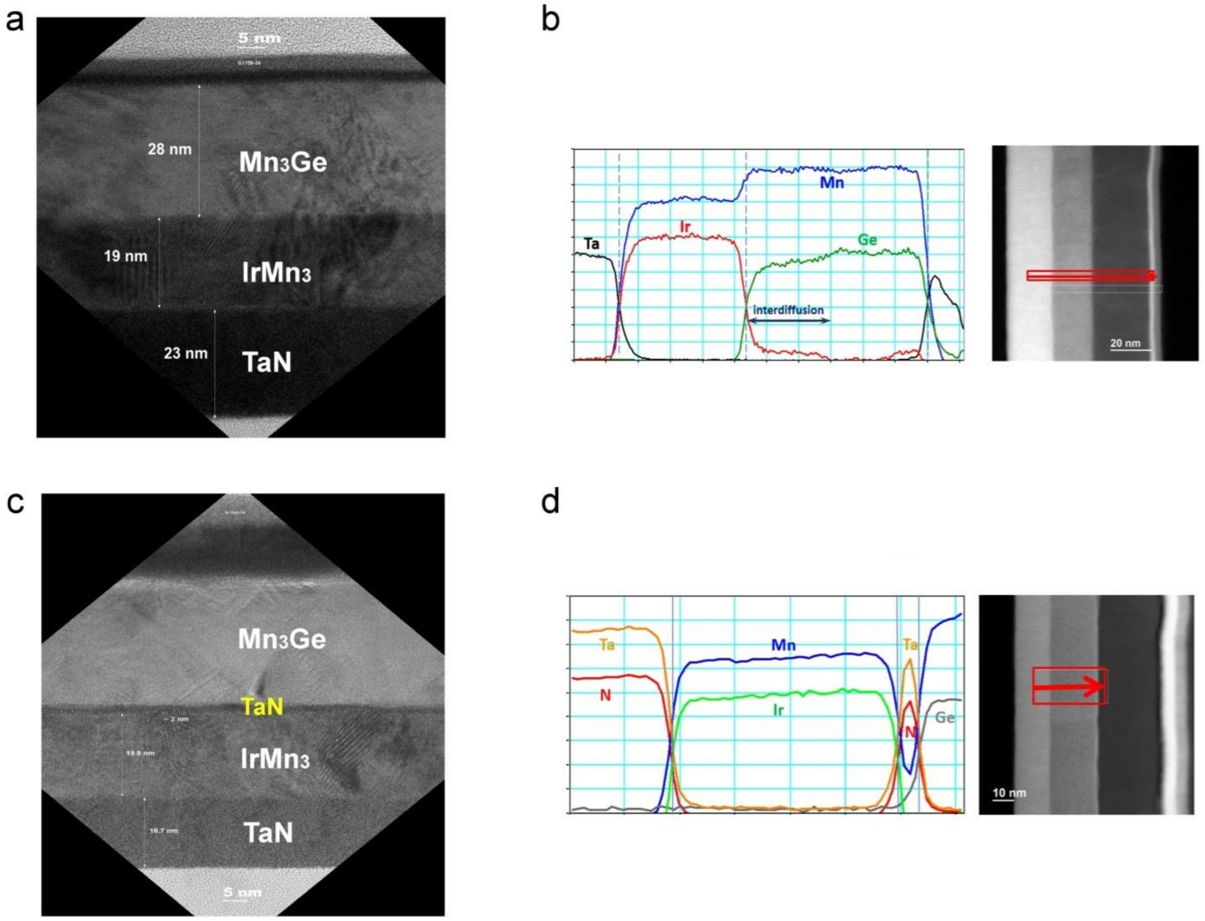

Supplementary Figure 3. HRTEM and EELS of Mn_3Ge films grown on TI (**a** and **b**) and TIT (**c** and **d**) underlayers. In **b** and **d** the spacing between two vertical lines corresponds to 5 nm.

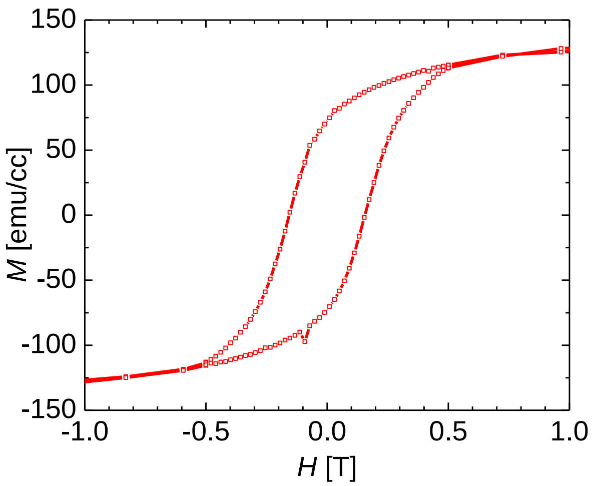

Supplementary Figure 4. *M* vs. *H* loop for a 300 Å Mn_2CuSb film grown at 450 °C on TIT underlayers. The measurement was carried out at RT.

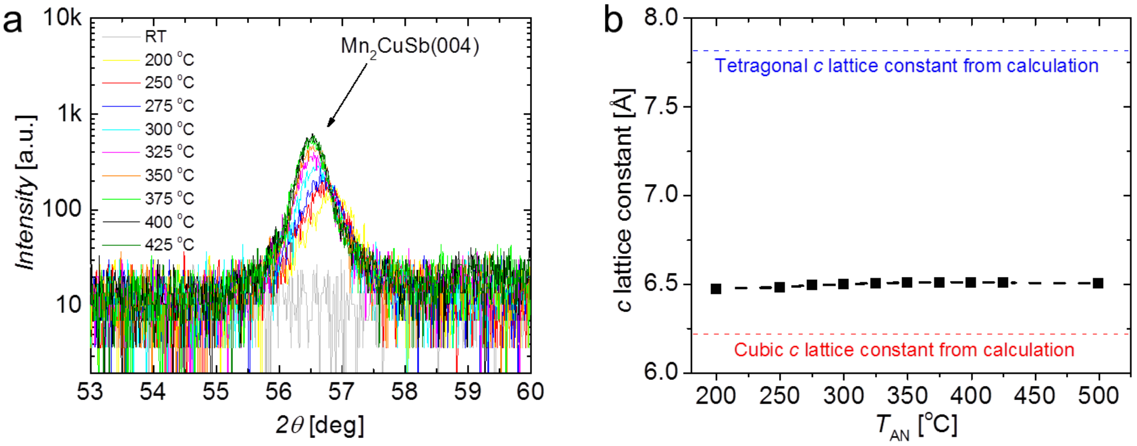

Supplementary Figure 5. a XRD measurements for a Mn_2CuSb film ex-situ annealed at different $T_{AN.}$ **b** Experimental (black squares) out-of-plane *c* lattice constants extracted from **a**.

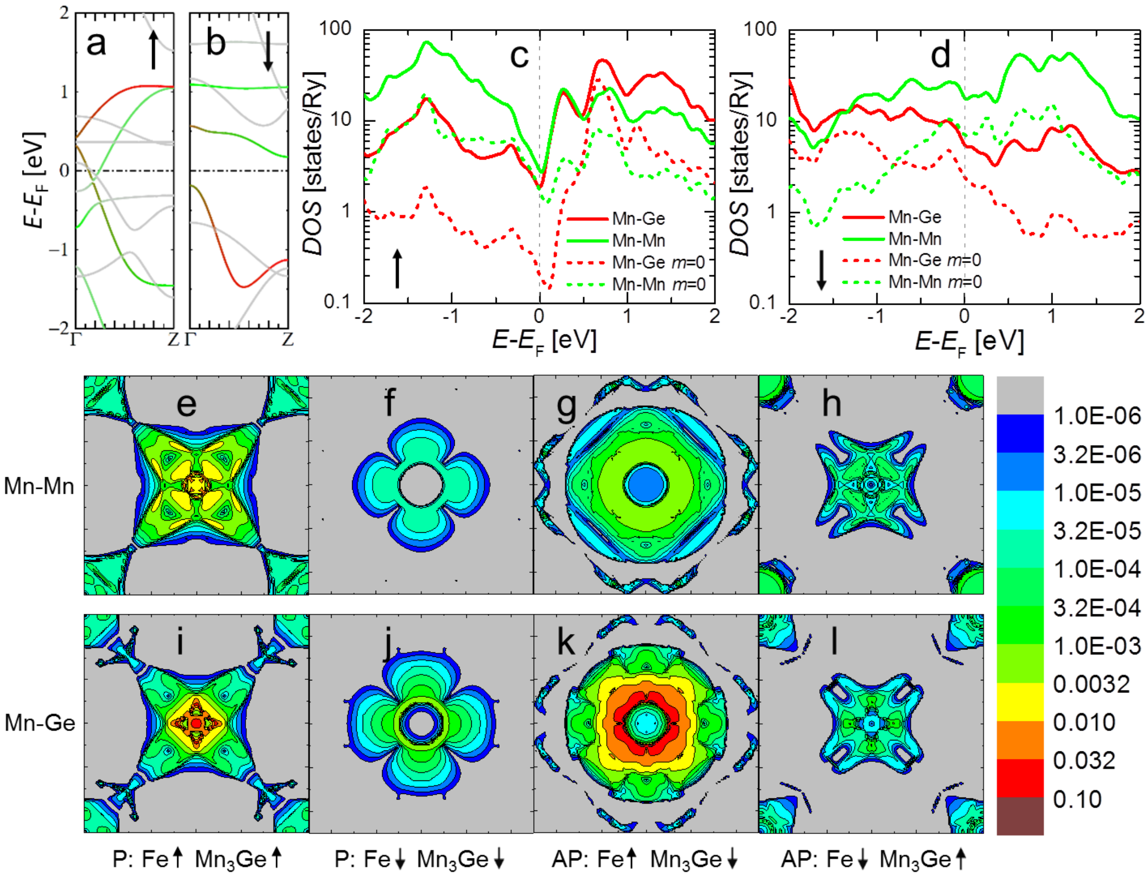

Supplementary Figure 6. Majority (**a**) and minority (**b**) electron bands of bulk Mn_3Ge along the Γ-Z line in the BZ. Majority (**c**) and minority (**d**) *DOS* of bulk Mn_3Ge projected to Mn-Ge and Mn-Mn layers (solid red and green lines, respectively) as well as to m = 0 basis orbitals of Mn-Ge and Mn-Mn layers (dashed red and green lines, respectively). $k_{||}$-resolved transmission functions for a Mn_3Ge/ MgO/ Fe MTJ calculated with N_{MgO} = 4 for both Mn-Mn (**e**, **f**, **g**, **h**) and Mn-Ge terminations (**i**, **j**, **k**, **l**) in P state (majority-Fe/majority-Mn_3Ge channel (**e** and **i**) and minority-Fe/minority-Mn_3Ge channel (**f** and **j**)), and AP state (majority-Fe/minority-Mn_3Ge channel (**g** and **k**) and minority-Fe/majority-Mn_3Ge channel (**h** and **l**)).

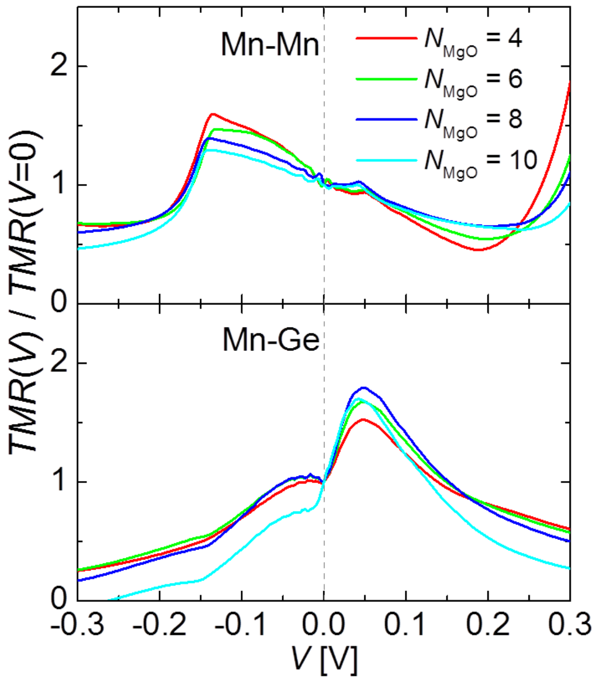

Supplementary Figure 7. The ratio of the optimistic *TMR*(*V*) calculated for a Mn_3Ge/ MgO/ Fe MTJ with Mn-Mn termination (**a**) and Mn-Ge termination (**b**) at bias voltage (-*V*) applied to the Fe electrode with respect to the *TMR* calculated at zero voltage *TMR*(*V*=0) for different N_{MgO}.

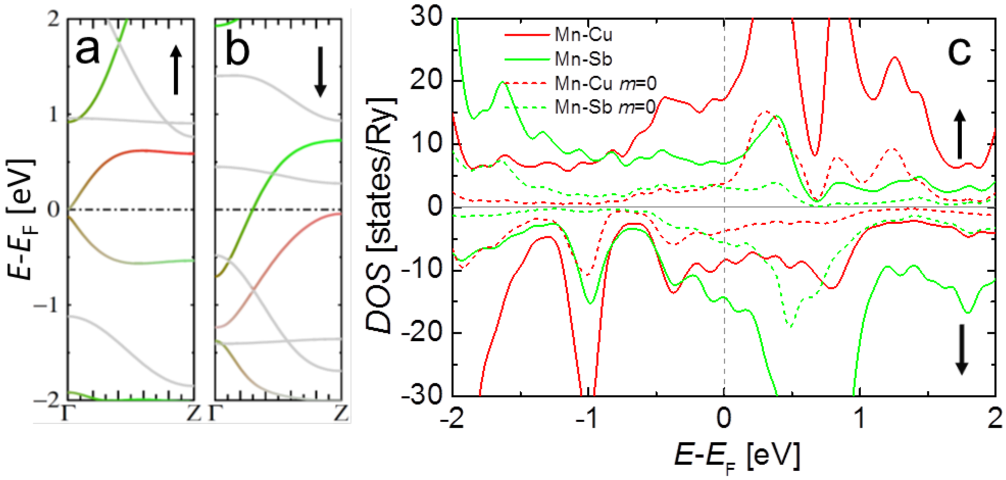

Supplementary Figure 8. Majority (**a**) and minority (**b**) electron bands of bulk Mn_2CuSb along the Γ-Z line in the BZ. **c** *DOS* of bulk Mn_2CuSb for majority (upper half) and minority (lower half) electrons projected to the Mn-Cu and Mn-Sb layers (solid red and green lines, respectively) as well as to $m = 0$ basis orbitals of Mn-Cu and Mn-Sb layers (dashed red and green lines, respectively).

Supplementary Note 1

TaN and $IrMn_3$ (TI) underlayers growth on Si(001)/ SiO_2 substrate

Supplementary Figure 1 shows x-ray diffraction (XRD) out-of-plane θ-2θ scans for TaN/ $IrMn_3$ bilayers deposited on Si(001)/ SiO_2 amorphous substrates. Ta_xN films were deposited with different Ar/N_2 ratios by reactive magnetron sputtering from a Ta target, and 200 Å $IrMn_3$ films were deposited using ion beam sputtering from a $IrMn_3$ target. These layers were grown at room temperature (RT). For the Ar/N_2 ratios within the range 95/5 – 75/25, all $IrMn_3$ films on Ta_xN had a preferential orientation of (001). $IrMn_3$ film grown on pure Ta underlayer showed a (111) orientation. Supplementary Figure 1b compares the orientation of 200 Å $IrMn_3$ films deposited on different thicknesses of TaN grown with a Ar/N_2 ratio of 90/10. At zero thickness (olive line) of TaN underlayer the $IrMn_3$ film had a (111) orientation, whereas all other films showed (001) orientation. We find that the $IrMn_3$(002) peak intensity increased with TaN thickness.

Supplementary Note 2

Growth of highly textured and smooth Mn_3Ge films on Si(001)/ SiO_2 substrates for magnetic tunnel junction electrode

Supplementary Figure 2a shows XRD out-of-plane θ-2θ scans for a series of 300 Å thick Mn_3Ge films deposited at increasing growth temperature (T_G). TaN (111) and $IrMn_3$ (002) peaks were observed at all T_Gs, though no distinct Mn_3Ge peak was detected for T_G = RT. Mn_3Ge superlattice (002) peaks were observed for films deposited at $T_G \geq 200$ °C.

Supplementary Figure 2b displays the quality factor, defined as the ratio $I(002)_{exp}/I(004)_{exp}$ of the XRD peak intensities of Mn_3Ge films (extracted from Supplementary Figure 2a), which is directly related to the chemical ordering of Mn and Ge in the Heusler alloy. The ratio $I(004)_{Mn3Ge}/I(002)_{IrMn3}$ (red open squares), the measure of texture of Mn_3Ge, increases with increasing T_G. Supplementary Figure 2b also shows the dependence of the root mean square roughness, r_{rms}, on T_G, measured by atomic force microscopy. We find that r_{rms} monotonically increased with T_G: r_{rms} < 5 Å for $T_G \leq 200$ °C, whereas r_{rms} > 20 Å for $T_G > 200$ °C, making these latter Mn_3Ge films unsuitable for use in technological applications. Smooth Mn_3Ge films (r_{rms} of ~3 Å) for MTJs grown using a 3-step (olive) process (as described in main text) display comparable chemical ordering as the films grown at T_G = 450 °C (Supplementary Figure 2b).

Supplementary Note 3

High-resolution transmission electron microscopy (HRTEM) and electron energy loss spectroscopy (EELS) of Mn_3Ge films with and without thin TaN diffusion barrier.

Cross-sectional HRTEM images of films with the structure Si/ 250 Å SiO_2/ 200 Å TaN/ 200 Å $IrMn_3$ (TI)/ 300 Å Mn_3Ge (grown at RT and in-situ annealed at 450 °C)/ 30 Å Ta, and Si/ 250 Å SiO_2/ 200 Å TaN/ 200 Å $IrMn_3$/ 20 Å TaN (TIT)/ 300 Å Mn_3Ge (3-step process)/ 30 Å Ta, are displayed in Supplementary Figure 3a and Supplementary Figure 3c with their corresponding EELS measurements in Supplementary Figure 3b and Supplementary Figure 3d, respectively. EELS data were collected across the samples region highlighted in the red horizontal rectangles (right panels), from left to right as indicated by the arrows. The concentration of Ta, N, Mn, Ir, and Ge was determined within the probed region. These data clearly indicate that Ir and Ge inter-diffuse for Mn_3Ge films on TI underlayers. No sign of inter-diffusion was found when the TaN diffusion barrier was deposited between the $IrMn_3$ and Mn_3Ge layers.

Supplementary Note 4

Magnetic property of Mn_2CuSb film deposited on amorphous Si(001)/ SiO_2 substrate with TaN/ $IrMn_3$/ TaN (TIT) underlayer.

The out-of-plane magnetization (*M*) vs. magnetic field (*H*) hysteresis loop for a Mn_2CuSb film deposited on TIT underlayer using Si(001)/ SiO_2 substrate is shown in Supplementary Figure 4. We find that the values of the saturation magnetization (M_S ~125 emu/cc) and coercive field (H_C ~1.5 kOe) are slightly higher than those found for the Mn_2CuSb film grown on single crystal MgO(001) substrate using 20 Å MgO/400 Å Cr (MC) underlayer (M_S ~70 emu/cc and H_C ~1 kOe). Nevertheless, MC underlayer was used to fabricate MTJ devices due to the squarer *M* vs. *H* loop and lower r_{rms} of these films compared to the TIT case (TIT : M_R/M_S ~0.58 and r_{rms} ~7 Å, MC : M_R/M_S ~0.93 and r_{rms} ~3.5 Å, M_R being the remanent magnetization).

Supplementary Note 5

Crystal structure dependence on ex-situ anneal temperature of a Mn_2CuSb film.

A 300 Å Mn_2CuSb film was grown at RT on MgO(001) single crystal substrate using MC underlayers, and capped with 30 Å Ta. The sample was then post-annealed at subsequent temperatures (T_{AN}) using a high-vacuum anneal chamber for half an hour in an applied (out-of-plane) magnetic field of 1 T.

Supplementary Figure 5a shows XRD out-of-plane θ-2θ scans for increasing T_{AN}. Here we only show the Mn_2CuSb (004) peak and have not included (002) and the forbidden (001) and (003) peaks. The Mn_2CuSb (004) peak become sharper and slightly shifts to smaller 2θ angles upon increasing T_{AN}, indicating improved tetragonality of the film. Supplementary Figure 5b compares the calculated regular tetragonal (blue dotted line), inverse cubic (red dotted line), and experimental *c* lattice constants (black squares). A ratio of $c_t/\sqrt{2}a_t$ ~1.1 (c_t and a_t being the out-of-plane and in-plane lattice constants of the tetragonal unit cell, respectively) is estimated from the Cr in-plane *a* lattice constant of 2.88 Å and experimental Mn_2CuSb *c* value of 6.5 Å, by assuming epitaxial growth of the Mn_2CuSb film on MC underlayer.

Supplementary Note 6

Increase of the *TMR* with increasing MgO thickness and opposite sign of the *TMR* for Mn-Ge and Mn-Mn terminations in Mn_3Ge/ MgO/ Fe MTJ

We first explain the increase of the *TMR* with increasing MgO thickness (N_{MgO}). The transmission (T) in a Mn_3Ge/ MgO/ Fe MTJ is dominated, for both parallel (P) and anti-parallel (AP) states (T_P and T_{AP} respectively), by majority Fe electrons. The contribution of minority Fe electrons to transmission is strongly suppressed due to the well-known symmetry spin filtering properties of the Fe/ MgO interface [1,2], as briefly described below.

In a Fe/ MgO/ Fe MTJ the MgO(001) spacer focuses the transmission function into the Γ-point in the $\mathbf{k}_{||}$ plane of the two-dimensional Brillouin zone (2D BZ), since in bulk MgO the evanescent state with $\mathbf{k}_{||} = 0$ has the smallest attenuation constant for energies within the MgO band gap. This state has Δ_1 symmetry (it is invariant with respect to the square-group symmetry transformations of x and y coordinates) and mostly consist of a mixture of $m = 0$ orbitals: *s*-orbitals of Mg and p_z-orbitals of O (m is the z-axis projection of the angular momentum). The attenuation constant increases as the in-plane momentum $\mathbf{k}_{||}$ increases. Therefore, as MgO thickness increases the transmission functions are focused closer and closer to the Γ-point in the 2D BZ[1,2].

In bulk Fe, the majority electrons have states with Δ_1-symmetry at the Γ-H line (along k_z-axis) in the BZ at the Fermi energy, E_F, therefore they can couple to the Δ_1-symmetry evanescent state of MgO. On the other hand, minority Fe electrons do not have bands with Δ_1-symmetry crossing the Γ-H line at E_F. Thus, due to symmetry mismatch, minority Fe electrons with $\mathbf{k}_{||} = 0$ cannot couple to the Δ_1-symmetry evanescent state of MgO suppressing their contribution to transmission at Fe/ MgO interface. This effect is called symmetry spin filtering[1,2]. In addition, bulk Fe has large positive spin polarization (spin polarization is defined as the ratio of the difference over sum of the majority and minority density of states (*DOS*) at E_F). Both symmetry spin filtering and large positive spin polarization of Fe contribute towards increase of the ratio T_P/T_{AP}. Hence, the *TMR* in a Fe/ MgO/ Fe MTJ is large and robust, with optimistic *TMR* > 10,000% for $N_{MgO} \geq 8$, as shown in Fig. 4a.

In Mn_3Ge/ MgO/ Fe and Mn_2CuSb/ MgO/ Fe MTJs the contribution to the transmission from majority Fe electrons is much larger compared to contribution from minority Fe electrons for the above reasons. Our calculations of transmission functions confirmed negligible contribution from minority Fe electrons in Mn_3Ge/ MgO/ Fe and Mn_2CuSb/ MgO/ Fe MTJs for both P and AP states, both terminations, and for all

N_{MgO} we studied (see, for example, the transmission function of the Mn_3Ge/ MgO/ Fe MTJ for $N_{MgO} = 4$ in Supplementary Figure 6).

Supplementary Figures 6a and 6b show the majority (**a**) and minority (**b**) electron bands of bulk Mn_3Ge along the Γ-Z line (along k_z-axis) in the BZ. The red, green and light-grey colors of the bands are mixed with weights proportional to the projection of the wave function of the band to specific orbitals as follow: red color represents the $m = 0$ basis orbitals centered at the Mn and Ge atoms of the Mn-Ge layer, green color represents the $m = 0$ basis orbitals centered at the Mn atoms of the Mn-Mn layer, and grey color refers to all remaining $m \neq 0$ basis orbitals. Only the majority electrons of bulk Mn_3Ge have states crossing the Γ-Z line at E_F, and some of these states have significant $m = 0$ weights (red and green). These states are not prohibited by symmetry considerations from coupling to the Δ_1 evanescent band of MgO.

Analogous to the symmetry filtering effect we refer to the combination of three factors - the absence of minority Mn_3Ge states near E_F along Γ-Z line, the presence of majority Mn_3Ge states crossing the Γ-Z line at E_F, and lastly the Γ-point focusing property of MgO spacer - as the Brillouin zone filtering effect[3]. For sufficiently large N_{MgO} the BZ filtering suppresses the contribution to transmission from minority Mn_3Ge electrons. Similarly to the symmetry filtering, the strength of the BZ filtering effect increases with N_{MgO}, since both effects are based on the Γ-point focusing property of MgO.

The transmission in a Mn_3Ge/ MgO/ Fe MTJ is dominated by majority Fe electrons in both P and AP states which means the dominant contribution to T_P and T_{AP} comes from majority and minority Mn_3Ge electrons, respectively. Since contribution to transmission from minority Mn_3Ge electrons is suppressed due to the BZ filtering effect, the ratio T_P/T_{AP} (hence *TMR*) should increase with N_{MgO}, in agreement with the *TMR* predictions displayed in Fig. 4a.

Supplementary Figure 6c and Supplementary Figure 6d show majority and minority electron *DOS* of bulk Mn_3Ge projected to Mn-Ge and Mn-Mn layers as well as to $m = 0$ basis orbitals of Mn-Ge and Mn-Mn layers. For both Mn-Ge and Mn-Mn layers the spin polarization is negative. Therefore, unlike the Fe/ MgO case where spin filtering and positive Fe spin polarization reinforce each other, in the Mn_3Ge/ MgO case the BZ filtering and negative spin polarization of Mn_3Ge oppose each other. BZ filtering suppresses the contribution to current from minority Mn_3Ge channel (which tends to make *TMR* of the Mn_3Ge/ MgO/ Fe MTJ positive) while negative spin polarization of Mn_3Ge enhances the contribution to the current from minority Mn_3Ge channel (which tends to make *TMR* of the Mn_3Ge/ MgO/ Fe MTJ negative). The sign of the *TMR* in Mn_3Ge/ MgO/ Fe system is determined by a delicate balance of these two opposing effects and is influenced by several factors such as MgO thickness, terminations at Mn_3Ge/ MgO interface and also applied bias voltage.

The evanescent state of bulk MgO with the smallest attenuation constant for states with fixed $k_{||}\neq 0$ still consists mostly of m = 0 orbitals (s-orbitals of Mg and p_z-orbitals of O) in the area of the 2D BZ close to the Γ-point. Thus, due to symmetry considerations, this evanescent state of MgO will couple more strongly to the m = 0 orbitals than to $m \neq 0$ orbitals of the termination layer of Mn_3Ge. Supplementary Figure 6c shows that for the Mn-Ge layer the majority *DOS* projected to m = 0 orbitals at E_F is roughly an order of magnitude smaller than the minority *DOS* projected to m = 0 orbitals at E_F, while for the Mn-Mn layer the *DOS* projected to m = 0 orbitals in majority and minority channel do not show such substantial differences. Thus, large difference in spin polarization of *DOS* projected to m = 0 orbitals in Mn-Ge and Mn-Mn layers favors negative *TMR* for Mn-Ge termination as compared to Mn-Mn termination for the same N_{MgO}. This conclusion is in agreement with the *TMR* presented in the main text (Fig. 4a). Indeed, Fig. 4a shows that for Mn-Ge termination the *TMR* is negative while for Mn-Mn termination *TMR* is positive (for $N_{MgO} > 2$). Since BZ filtering effect becomes stronger as N_{MgO} increases, the *TMR* in the case of Mn-Ge termination becomes less negative and, at sufficiently large N_{MgO}, it will eventually change sign and become positive.

Supplementary Note 7

Bias voltage dependence of *TMR* in a Mn_3Ge/ MgO/ Fe MTJ for Mn-Mn and Mn-Ge terminations.

In Supplementary Figure 7, we present the ratio between the optimistic *TMR*(V) calculated at finite bias voltage equals to (-V) (with $|V| < 0.3$ V) applied to the Fe electrode in Mn_3Ge/ MgO/ Fe MTJ, to the *TMR* calculated at zero voltage, *TMR*(V = 0). Note that, because of the negative charge of the electron, e, we define the voltage applied to the Fe electrode as (–V), in order to induce a positive shift of the Fe Fermi energy, $E_F + e(-V)$, relative to the Mn_3Ge Fermi energy, E_F, for positive V. For evaluation of the *TMR*(V) we use the following approximation for the current I:

$$I = \frac{e}{h} \int_{E_F}^{E_F - eV} T(E)dE \tag{1}$$

where $T(E)$ is the equilibrium transmission function of the corresponding spin channel calculated at $V = 0$ and E_F is the Fermi energy of the Mn_3Ge electrode. As described above, in a Mn_3Ge/ MgO/ Fe system the dominant contribution to transmission both in P state, T_P, and in AP state, T_{AP}, derives mainly from the Fe majority channel. Since majority-majority transmission in a Fe/ MgO/ Fe system is flat and featureless (at least within 0.3 eV from the Fe E_F), the shape of the transmission function of the Mn_3Ge/ MgO/ Fe MTJ for $|E - E_F| < 0.3$ eV is determined by the energy-dependent features at the Mn_3Ge/ MgO interface. Hence

the current at voltage (-V) applied to Fe can be approximated by integrating the equilibrium transmission $T(E, V = 0)$ from the Fermi energy of the Mn_3Ge electrode, E_F, to the Fermi energy of the Fe electrode, E_F +e(-V).

For Mn-Mn termination $I_P > I_{AP}$ (considering $N_{MgO} > 2$), so the optimistic $TMR(V) = I_P/I_{AP} – 1$. The sharp drop of $TMR(V)$ for Mn-Mn termination at V = -0.15 V (Supplementary Figure 7a) can be attributed to an increase of the T_{AP} at E_F - 0.15 eV due to the appearance of the minority electron band of Mn_3Ge at the Γ-Z line (see Supplementary Figure 6b). In other words, there is no more BZ filtering effect at $E < E_F - 0.15$ eV. The minimum of the $TMR(V)$ at 0.2 V can be attributed to the minimum in DOS of the majority Mn_3Ge electrons at E_F + 0.15 eV (see Supplementary Figure 6c) resulting in an increase of T_P at $E > E_F +$ 0.15 eV.

For Mn-Ge termination, $I_P < I_{AP}$ so the optimistic $TMR(V) = 1- I_{AP}/I_P$. The shape of $TMR(V)$ for Mn-Ge termination is mostly determined by the shape of $T_{AP,}$ that has a maximum at E_F + 0.05 eV. We find that at $N_{MgO} = 10$ the TMR changes sign for V = -0.27 V confirming that the delicate balance between the two opposing effects - BZ filtering and negative spin polarization of Mn_3Ge - can be affected not only by switching the terminations or increasing the MgO thickness, but also by applying relatively small bias voltage.

Supplementary Note 8

Opposite sign of *TMR* for Mn-Cu and Mn-Sb terminations in a Mn_2CuSb/ MgO/ Fe MTJ.

In Supplementary Figure 8 we present majority (**a**) and minority (**b**) electron bands of bulk Mn_2CuSb along Γ-Z line (along k_z-axis) of the BZ. The red, green and light-grey colors of the bands are mixed with weights proportional to the projection of the wave function of the band to specific orbitals as follow: red color represents the $m = 0$ basis orbitals centered at the Mn and Cu atoms of the Mn-Cu layer, green color represents the $m = 0$ basis orbitals centered at the Mn and Sb atoms of the Mn-Sb layer, and grey color refers to all remaining $m \neq 0$ basis orbitals. Supplementary Figure 8b shows that only in minority Mn_2CuSb channel there is a band with green color that crosses the Γ-Z line at E_F. We assume that at Mn_2CuSb/ MgO interface the coupling of the Δ_1-symmetry evanescent state of (bulk) MgO with $\mathbf{k}_{\|} = 0$ to the band of (bulk) Mn_2CuSb crossing the Γ-Z line at E_F increases with the weight of the projection of this Mn_2CuSb band to the $m = 0$ basis orbitals centered at the atoms of the termination layer. In case of Mn-Sb termination the $\mathbf{k}_{\|} = 0$ Δ_1-symmetry evanescent state of MgO couples more strongly with minority Mn_2CuSb channel. Therefore, at sufficiently large MgO thickness, when transmission functions are

concentrated near the $\mathbf{k}_{||} = 0$ point we expect a negative *TMR* for a Mn_2CuSb/ MgO/ Fe MTJ with Mn-Sb termination, in agreement with the calculated *TMR* presented in Fig. 4c. In addition, the spin polarization of the Mn-Sb layer based on both total and $m = 0$ *DOS* is negative thus favoring negative *TMR* even at small MgO thicknesses, which is again in agreement with the *TMR* presented at Fig. 4c.

For Mn-Cu termination, the red colored band in the majority channel (Supplementary Figure 8a) crossing the Γ-Z line at E_F has a large grey ($m \neq 0$) component at E_F. In the minority channel (Supplementary Figure 8b) the red colored band is very close to E_F and hence, after integration over $k_{||}$, gives a contribution comparable as if it was actually crossing the Γ-Z line at E_F. Therefore we cannot determine the sign of the *TMR* for Mn-Cu termination based on the band structure along the Γ-Z, though the *TMR* will likely be positive (since the red majority band really crosses E_F even though it has a large grey component). The spin polarization of the Mn-Cu layer based on both total and $m = 0$ *DOS* is positive (Supplementary Figure 8c and 8d), thereby favoring positive *TMR*. We expect positive *TMR* for a Mn_2CuSb/ MgO/ Fe MTJ with Mn-Cu termination, though its absolute value will be smaller compared to *TMR* with Mn-Sb termination, which again is in agreement with the calculated *TMR* displayed in Fig. 4c.

Note that the arguments concerning the sign of the *TMR* presented in this and previous sections only take into account the bulk properties of the Heusler compound and do not include, for example, interface-related effects (e.g. presence of interface resonance states, shift of potential for interface atoms, etc.) that can also affect the magnitude and even the sign of the *TMR*. The arguments we have used are useful for the selection of promising candidate materials from the large family of Heusler compounds. More precise calculations of the electronic structure and transmission functions for the complete MTJ system will lead to more reliable values of the *TMR*.

Supplementary References

1 Butler, W. H., Zhang, X.-G., Schulthess, T. C. & MacLaren, J. M. Spin-dependent tunneling conductance of Fe|MgO|Fe sandwiches. *Phys. Rev. B* **63**, 054416, (2001).

2 Mathon, J. & Umerski, A. Theory of tunneling magnetoresistance of an epitaxial Fe/MgO/Fe(001) junction. *Phys. Rev. B* **63**, 220403, (2001).

3 Faleev, S. V., Parkin, S. S. P. & Mryasov, O. N. Brillouin zone spin filtering mechanism of enhanced TMR and correlation effects in Co(0001)/h-BN/Co(0001) magnetic tunnel junction. Preprint at http://arxiv.org/abs/1504.01017 (2015).
